# Covert Waking Brain Activity Reveals Instantaneous Sleep Depth

**DOI:** 10.1371/journal.pone.0017351

**Published:** 2011-03-03

**Authors:** Scott M. McKinney, Thien Thanh Dang-Vu, Orfeu M. Buxton, Jo M. Solet, Jeffrey M. Ellenbogen

**Affiliations:** 1 Department of Neurology, Massachusetts General Hospital, Boston, Massachusetts, United States of America; 2 Division of Sleep Medicine, Harvard Medical School, Boston, Massachusetts, United States of America; 3 Cyclotron Research Centre, University of Liege, Liege, Belgium; 4 Department of Medicine, Brigham and Women's Hospital, Boston, Massachusetts, United States of America; 5 Department of Medicine, Cambridge Health Alliance, Cambridge, Massachusetts, United States of America; Central Queensland University, Australia

## Abstract

The neural correlates of the wake-sleep continuum remain incompletely understood, limiting the development of adaptive drug delivery systems for promoting sleep maintenance. The most useful measure for resolving early positions along this continuum is the alpha oscillation, an 8–13 Hz electroencephalographic rhythm prominent over posterior scalp locations. The brain activation signature of wakefulness, alpha expression discloses immediate levels of alertness and dissipates in concert with fading awareness as sleep begins. This brain activity pattern, however, is largely ignored once sleep begins. Here we show that the intensity of spectral power in the alpha band actually continues to disclose instantaneous responsiveness to noise—a measure of sleep depth—throughout a night of sleep. By systematically challenging sleep with realistic and varied acoustic disruption, we found that sleepers exhibited markedly greater sensitivity to sounds during moments of elevated alpha expression. This result demonstrates that alpha power is not a binary marker of the transition between sleep and wakefulness, but carries rich information about immediate sleep stability. Further, it shows that an empirical and ecologically relevant form of sleep depth is revealed in real-time by EEG spectral content in the alpha band, a measure that affords prediction on the order of minutes. This signal, which transcends the boundaries of classical sleep stages, could potentially be used for real-time feedback to novel, adaptive drug delivery systems for inducing sleep.

## Introduction

Sleep is not uniform, and certain moments are sounder than others. Indeed, resistance to acoustic disturbance—a measure of sleep depth—displays considerable variability throughout a night of sleep, even within sleep stage [1]. The factors that influence sleep's vulnerability to sensory insult have not been fully illuminated.

**Figure 1:**
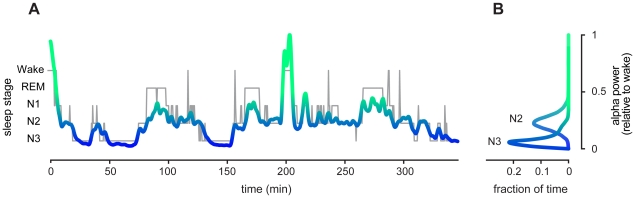
Alpha power fluctuates dynamically throughout the night. A. The trajectory of relative alpha power throughout a quiet night of sleep is shown from one representative subject. Simultaneous sleep stage designations run beneath the time course of alpha power. Diminishing as sleep begins, alpha power fluctuates throughout the night, in tandem with sleep depth. For display, the alpha power time-series was approximated using local linear regression in 4 minute windows, corresponding to the approximate length of each stimulation series (see Materials and Methods). This procedure removes noise and emphasizes slower fluctuations with fewer distortions than those imposed by simple low-pass filtering [42]. B. These histograms show the distribution of alpha power during NREM sleep, revealing that a range of values can be observed within each of stages N2 and N3. Power was computed in non-overlapping 10-second bins; epochs containing arousals, which may represent transient departures from stable sleep, were discarded.

The very transition from wake to sleep involves a dissociation from the external world and a crescendo of internal brain rhythms. Heralding this transition is attenuation of the alpha rhythm, an 8–13 Hz electroencephalographic (EEG) oscillation prominent over posterior brain regions, and the signature of relaxed wakefulness [2]. Diminishing during the descent into sleep, alpha amplitude shadows the decline in external awareness that accompanies sleep onset [3], [4]. And while it appears to vanish as sleep begins, quantitative analysis reveals that power in the alpha band actually fluctuates dynamically throughout the night (Fig. 1A) [5].

Given alpha activity's association with wakefulness and sensory intake, we hypothesized that covert levels of alpha activity would reveal a sleeper's instantaneous sensitivity to the environment. That is, inconspicuous fluctuations in wake-like background brain activity might correspond to changes in sleep depth, even beyond sleep stage designation.

To study this question in a realistic setting, we used ecological noises to probe environmental sensitivity throughout sleep, simultaneously monitoring subjects' brain activity with EEG. The sound intensity required to disturb subjects provided an empirical measure of their instantaneous sleep depth. In this paradigm, *sleep stability* denotes resistance to disruption, while *sleep fragility* denotes vulnerability to disruption. We sought to evaluate whether these qualities could be predicted using the covert level of waking brain activity just before each stimulus.

## Results

**Figure 2:**
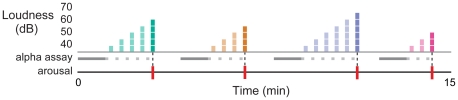
Sleep depth was probed with auditory stimulation. We systematically probed sleep depth with auditory stimulation during bouts of N2, N3 and REM sleep. Ten-second noises were initiated at 40 decibels (dB) and presented every thirty seconds in 5 dB increments until the EEG signal was perturbed (arousal, vertical bars on the bottom line). Each color represents a different sound type; a sample of four is shown here. The sound intensity required to disturb subjects provided an experimental measure of their immediate sleep depth. The gray windows beneath the sound level delineate periods during which alpha power was measured to predict sleep fragility.

We systematically challenged sleep with auditory stimulation in thirteen healthy subjects throughout two nights of sleep. Brain activity was monitored on each night using EEG. Ten-second, ecological noises (e.g., road and air traffic, a telephone ringing) were presented during bouts of stable sleep (Fig. 2). Each sound was initiated at 40 decibels (dB) and replayed every thirty seconds in 5 dB increments until the EEG signal was perturbed according to standard guidelines (i.e., an arousal was observed [6]).

We interrogated the relationship between alpha activity and sleep fragility using Cox regression, a tool from survival analysis (see Materials and Methods). The output of Cox regression is the hazard ratio (HR): this number represents the relative hazard of disruption in one condition compared to another. For continuous covariates, the HR represents the relative hazard of disruption incurred by a one-unit increase of the covariate. Hazard ratios greater than 1 imply that the covariate is associated with sleep fragility (vulnerability to disruption), while those less than 1 denote covariates accompanying sleep stability (resistance to disruption).

We focused our analysis on factors contributing to sleep fragility during non-rapid-eye-movement (NREM) sleep (stages N2 and N3, accounting for the majority of sleep [7]), as several difficulties arise when considering alpha activity during rapid-eye-movement (REM) sleep (see Discussion). The regression model contained two covariates, one indicating the visually scored sleep stage designation [6], the other indicating the spectral content preceding each stimulus (see Materials and Methods).

When comparing noise sensitivity across sleep stage, Cox regression yielded a HR of 0.54 (*P*<0.0001) associated with stage N3, so-called slow-wave sleep, relative to N2. In line with previous reports [1], [8], this value indicates a suppressed hazard of disruption in N3 compared with N2 (the probability of tolerating noise at any loudness in N3 being roughly the square root of that in N2).

We next addressed the influence of occipital alpha activity on sleep fragility. Figure 1A shows that, on an undisturbed night of sleep, relative alpha power shadows the trajectory of qualitatively assessed sleep depth. Like the probability of disruption, alpha power is generally suppressed in N3 relative to N2 (Fig. 1B).

Although relative alpha power correlates well with sleep stage, a wide spectrum of variation still exists within each category. We therefore sought to determine whether fluctuations in this quantity—even within sleep stage—correspond to concurrent variations in sleep fragility.

To address this question, we included in the statistical model a measure of the alpha content during the ten seconds immediately preceding each sound (Fig. 2, light gray windows). Even controlling for stage, we observed a highly significant relationship between alpha power and sleep fragility (HR = 5.74, *P*<0.001). This suggests that, well beyond sleep stage designation, latent alpha content betrays heightened sensitivity to impending sounds.

To investigate the timescale over which alpha power predicts sleep fragility, we further characterized each sound series by a single spectral measure derived from a reference window of stable sleep preceding the sound series (Fig. 2, dark gray windows). This interval anticipated the eventual disruption by a variable latency of up to four minutes, as arousal may have occurred as late as 70 dB. Still, alpha power during this ninety-second baseline period predicted the probability of disruption in the moments that followed (HR = 7.33, *P*<0.001), suggesting that the brain state disclosed by alpha activity persists for several minutes (see also Results S1 and Figure S1).

**Figure 3:**
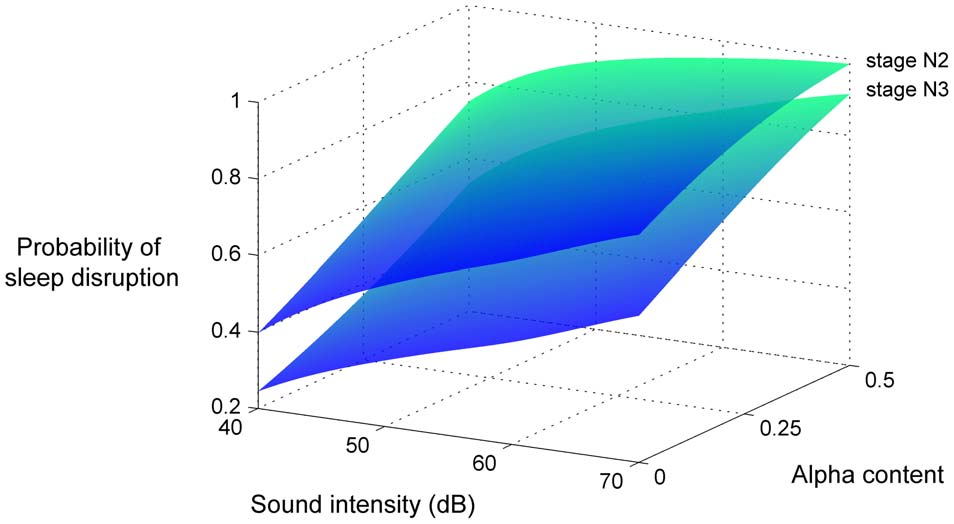
Alpha content reveals immediate sleep fragility. Using Cox regression, we found that alpha activity disclosed noise sensitivity during NREM sleep, even when controlling for stage. Here we reconstruct probability surfaces for sleep stages N2 and N3, rendering sleep fragility as a function of both stimulus intensity and EEG alpha content.

Figure 3 illustrates a summary of our results, rendering the probability of sleep disruption in the face of noise as a function of both stimulus intensity and EEG alpha content. Here we depict a distinct surface for each NREM sleep stage, in which separate mechanisms may also regulate sensory perception [9], [10]. Just as the probability of disruption increases monotonically with loudness, so too is sleep's vulnerability modulated by coincident alpha content.

We next explored the relationship between immediate sleep fragility and the broader EEG power spectrum. Toward this end, we estimated the power at frequencies between 0.5 and 25 Hz (in 0.5 Hz intervals) expressed over occipital electrodes during the ten seconds immediately preceding each stimulus (Fig. 2, light gray windows). To facilitate a meaningful comparison across frequencies, power values were standardized based on their waking levels and their dynamic ranges observed during NREM sleep (see Materials and Methods). The power at each frequency was then analyzed independently using Cox regression.

**Figure 4:**
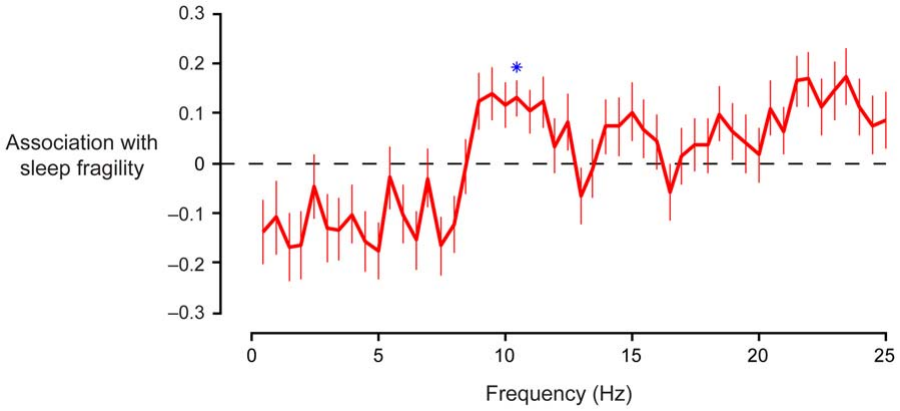
Alpha band power is a specific marker of sleep depth. We examined the broader relationship between EEG spectral content (beyond sleep stage designation) and NREM sleep depth. The power expressed over occipital electrodes immediately preceding each stimulus was standardized using a baseline waking spectrum and the dynamic range observed during sleep at each frequency. The relationship between spectral power values and sleep fragility was then assessed independently with Cox regression; the resulting regression coefficients (±SE) are shown for each frequency in 0.5 Hz bins. Coefficients less than zero imply an association with sleep stability (resistance to disruption), whereas coefficients greater than zero imply an association with sleep fragility (vulnerability to disruption). We observed a strong relationship between heightened sound sensitivity and spectral power throughout the alpha band. Trends toward sleep stability emerged in conjunction with low frequency (<8 Hz) power and toward fragility in conjunction with high frequency (>13 Hz) power. *P<0.05 after correction for multiple comparisons.

The resulting spectral portrait shows how power at each frequency, beyond stage designation, covaries with sleep fragility (Fig. 4). (For comparison across the entire EEG spectrum, estimates of the Cox regression coefficients reflect a change of one standard deviation in the log-power at each frequency.) The large, sustained contribution throughout the alpha band suggests that this region of the spectrum indeed contains a meaningful signal. We moreover observed strong tendencies toward sleep stability in conjunction with low-frequency power (including slow-wave, delta, and theta activity) and toward fragility in conjunction with high-frequency power (beta activity). Nonetheless, the only power value that achieved significance after a liberal correction for multiple comparisons (Holm-Bonferroni method) was that at 10.5 Hz (*P* = 0.025), centrally located within the alpha band.

As the approach just described manages to isolate the power at different frequencies, we took the opportunity to once more study the relationship between alpha activity and sleep fragility, this time including in our statistical model, in place of sleep stage designation, a measure of low-frequency oscillatory EEG activity (0.5–4 Hz), which may more faithfully track changes in sleep depth at the neuronal level [11]. This moreover teases apart the effects of alpha and delta activity, which may interact in the relative measure of alpha content employed earlier. In this context, slow-wave activity was associated with sleep stability (HR = 0.73; *P*<10^−11^), and alpha activity again demonstrated a significant relationship with sleep fragility (HR = 1.13; *P* = 0.002).

## Discussion

The present results show that the soundness of sleep, defined empirically and with ecological relevance, is revealed in real-time by EEG spectral content: greater vulnerability to noise-induced sleep disruption accompanies elevated alpha activity. Such spectral interrogation of sleep fragility has predictive power on the order of minutes. Furthermore, this effect transcended traditional sleep staging, imparting a greater sense of fluidity to what is typically seen as a rigid process.

From a behavioral perspective, alpha activity has been shown to resolve fine gradations in the sleep-wake continuum. On visual and auditory vigilance tasks, reduced alpha activity is associated with sluggish reaction times and an elevated probability of lapse [12], [13], [14], [15], [16]. Even during wakefulness, immediate levels of alertness are revealed by ongoing alpha activity: moments of higher parietal alpha amplitude have been associated with receptiveness to tactile stimuli and heightened attention [17]. These observations concerning alpha's relationship to sensory intake, in conjunction with several others, have emboldened some investigators to include the alpha oscillation among the neural correlates of consciousness [18]. Here we extend alpha activity's association with environmental awareness beyond wakefulness and drowsiness, and into NREM sleep.

Though the alpha oscillation was one of the first brain rhythms to be described in the human EEG [19], little is currently understood about its underlying generators or functional significance. The thalamus, which has been found to influence cortical alpha synchronization [20], might be the critical link between alpha activity and the brain's vulnerability to acoustic disruption. As the thalamus is involved in relaying sensory information to the cortex, alpha activity could be a reflection of this region's propensity for conveying external stimuli to cortical processing centers where it is capable of interrupting sleep. Intriguingly, it was recently shown that global expression of alpha power (and, to a weaker extent, beta power) is positively correlated with activity in a “tonic-alertness network,” comprised of the dorsal anterior cingulate cortex, anterior insula, and thalamus [21]. The constituents of this network, with access to sensory information and broad projections throughout the cortex [22], are well positioned to support alerting functions and a “general readiness for perception and action” [21], [23]. At least during wakefulness, then, elevated alpha activity seems to reflect the engagement of regions supporting sensory intake and alertness. Future studies should address the existence of a similar intrinsic connectivity network during sleep, and its connection with EEG alpha content.

### The specificity of alpha power as a maker of NREM sleep depth

When we broadened the scope of our analysis to include the rest of the EEG power spectrum during NREM sleep, only an alpha frequency (10.5 Hz) remained significant after correction for multiple comparisons. Rather than suggesting that this lone frequency contains information regarding sleep fragility, we suspect that inter-individual variability in peak alpha rhythm frequency [24] undermined the effect when small slivers of the band were considered alone.

Although large scale associations between EEG power and sleep depth might be expected based on inherent correlations across frequencies in the power spectrum of the sleeping brain [5], we nonetheless observed several trends outside the alpha band that warrant attention. In particular, we noted a tendency toward sleep stability in conjunction with increased power in the frequencies below 8 Hz. This accords well with the view that low-frequency oscillatory activity (including slow-wave and delta activity) intensifies with increasing depth of NREM sleep [11]. It should be noted, however, that our analysis controlled for NREM sleep stage, so the overall relationship between sleep stability and low-frequency power, which is enhanced in stage N3 relative to N2, was necessarily blunted in this context. The association between reduced sound sensitivity and EEG spectral power in the low frequencies appeared to extend even to the theta band (4–7 Hz). In light of this observation, it is interesting to note that during vigilance tasks, theta-rich EEG has been found to be associated with reduced arousal [25] and deteriorated stimulus detection [12], [26].

We further observed a tendency for increased vulnerability to disruption in conjunction with greater EEG power in higher frequencies, including the beta band (15–25 Hz). As with alpha activity, previous work has also shown a connection between variation in beta activity and fluctuations in cortical arousal and vigilance behavior [27], [28]. A similar observation was made in sleep, with enhanced beta activity now thought to signify heightened arousal in patients with insomnia [29].

As might be expected, the association between alpha power and sleep fragility did not yield significance when REM sleep was considered alone (*P* = 0.45). During REM, the relative alpha power is more erratic and this activity may stem from heterogeneous brain sources. Alpha activity during REM occurs in at least two forms, conspicuous alpha bursts and background alpha activity, which are thought to be electrophysiologically distinct from one another and from that evident during wakefulness. Further, alpha amplitude may be modulated by visual imagery in the context of dreaming [2]. While the function of alpha activity during REM remains hypothetical, the present results suggest that it does not accompany heightened sensitivity to one's environment.

### Future directions and applications

Previous electrophysiological studies have demonstrated that ongoing network activity profoundly influences evoked cortical responses and explains their dramatic variability [30]. Here we further emphasize the role of the immediate brain state in modulating perception by showing that beyond sleep stage [8] and the overt rhythms of sleep [31], inconspicuous background activity also varies with the soundness of sleep. In this light, alpha activity provides a potent window onto the instantaneous responsiveness of the sleeping brain. Future research should investigate the extent to which other features of EEG dynamics, such as spectral coherence [32], [33], cross-frequency phase synchrony [34], [35], or nested oscillations [36], [37] offer useful information about empirical measures of sleep depth.

Given that real-time fluctuations in EEG parameters provide immediate information about sleep's depth and its vulnerability to disruption, it is enticing to speculate that this kind of information could be employed by adaptive hypnotic agents guided by direct feedback from neural activity. Such technology might be capable of combating the disruptive effects of environmental noise on sleep and next-day cognitive performance [38], [39], while optimally preserving natural sleep physiology. At present, sleep medications are a blunt instrument. Administered before bed, conventional hypnotics last for a rigid duration fixed by their pharmacokinetic properties. These drugs dominate consciousness, inducing sleep-like sedation of unclear authenticity [40], [41]. A system that allows for dynamic drug delivery based on instantaneous feedback (using a metric derived from alpha activity or the broader EEG power spectrum) could momentarily protect or facilitate sleep when vulnerable, otherwise letting natural brain rhythms run their course. Further, such an arrangement might allow for emergency interruptions or scheduled wake-times; such specificity is prohibited by the crude sleep medications used today. Besides using smaller doses, then, this system would afford enhanced precision and flexibility. The present study establishes a conceptual framework for such research, showing that sleep can be monitored in real-time and characterized along a rich continuum of depth.

## Materials and Methods

The findings described here stem from an experiment conducted to study the disruptive salience of different sounds in sleep. Biomarkers for individual noise tolerance (i.e., traits) were presented in [10], whereas the current analysis seeks to elucidate moment-to-moment variations in sleep's vulnerability to disruption (i.e., states).

### Ethics statement

Study procedures were approved by the Human Research Committees of the Brigham and Women's Hospital, the Massachusetts General Hospital (MGH), and the Cambridge Health Alliance. Written informed consent was obtained for all participants.

### Participants

Thirteen healthy volunteers (9 females and 4 males, age 24.9±7.3; mean ± SD) were determined to be free from medical or psychiatric conditions on the basis of clinical history and a physical examination. Participants were also screened for drug, alcohol, or caffeine dependency. Subjects reported taking no medications that affect sleep or circadian rhythms. All participants demonstrated normal hearing on the basis of audiometric screening of each ear (minimum hearing level of 25 decibels [dB] at 500, 1000, 2000 and 4000 Hz).

### Study conditions

Participants slept on a consistent schedule for at least 4 days prior to the study, as confirmed by wrist actigraphy (AW-64, Minimitter, Bend, OR). During the study, subjects stayed at the MGH Sleep Laboratory for 3 consecutive nights. Each night, subjects were given the opportunity to sleep for 8.5 hours at their normal bedtime. Research staff monitored the subjects 24 hours a day to ensure that they did not nap. Light levels were maintained at approximately 90 lux during waking periods, and <1 lux during sleep periods. The first night was used for adaptation; subjects adjusted to the laboratory environment and were screened for any sleep disorders visible on the polysomnogram. Acoustic stimulation was applied only on the second and third nights.

### Sleep recordings

Polysomnographic recordings were collected using a Comet XL system (Grass-Telefactor, West Warwick, RI, USA). Skin surface electrodes (Beckman Instrument Company, Schiller Park, IL) captured EEG from frontal (F3 and F4), central (C3 and C4) and occipital (O1 and O2) positions; electrooculogram (EOG); submental electromyogram (EMG); and electrocardiogram (ECG). Data were conditioned by analogue filters (high-pass: 0.3 Hz; low-pass: 70 Hz), and digitally sampled at 200 Hz.

### Experimental paradigm

On the second and third nights of the experiment, acoustic stimulation was applied systematically throughout stages N2, N3 and REM sleep. Once stable sleep was achieved (at least 90 consecutive seconds of the same stage scored in real time), sounds were initiated at 40 dB and replayed every thirty seconds in 5 dB increments until an arousal was observed or 70 dB was reached (Fig. 2). A 70 dB limit was imposed to minimize full awakenings from sleep and prevent significant disruption of sleep architecture. Each time an arousal was elicited, sound was withheld until stable sleep resumed, at which time a new sound was chosen.

Acoustic stimuli were each ten seconds in duration, and drawn from diverse sources. Noises included a telephone ringing, a toilet flushing, an IV alarm, a hospital intercom, a door creaking and slamming, a laundry machine, an ice machine, a towel dispenser, road traffic, snoring, a jet engine, a helicopter, and two conversations of positive and negative emotional valence. All sounds except the jet and helicopter were recorded on site in a medical unit of Somerville Hospital, Somerville, MA. Stimuli, which were repeated through each graduated sound series, were selected at random for each participant on each night.

Sound levels were measured using dBA-L_eq-10 s_, consistent with standard methods used to evaluate the clinical effects of noise. “A” refers to the weighting of sounds in ranges audible to humans, while “L_eq-10 s_” denotes an average intensity derived from the 10 seconds of the sound's duration. The sound level in the patient room was logged with an environmental sound monitor (Rion Type NL-31, with Type 1 microphone) located 10 inches above the subject's head. Stimuli were presented on a measured average background of 34–35 dB due to continuous ventilation in the room.

Stimuli were delivered in surround sound using an array of four studio-monitor loudspeakers (Event, model PS6) placed at the circumference of a circle centered around the subject's head. This arrangement enabled sounds with moving sources (e.g., the airplane) to be reproduced with apparent motion through space.

### Sleep scoring

Sleep stages (in 30-second epochs) and arousals were identified in adherence with the recommendations of the American Academy of Sleep Medicine [6]. According to these criteria, an arousal consists of an abrupt increase in EEG frequency lasting at least 3 seconds, excluding that caused by a spindle, and preceded by at least 10 seconds of stable sleep. Sleep scoring was conducted by a registered polysomnographic technician under the supervision of the medical director of the MGH Sleep Laboratory.

### Spectral estimation

The period preceding sound presentation was used to assay the electroencephalographic sleep depth associated with each sound. Power spectra were estimated using the multitaper method [42]. Spectra were derived from occipital electrodes (average of O1 and O2), as waking alpha tends to predominate over these posterior regions [43].

For each segment of analysis, alpha activity was computed as the integral of the power spectrum in the alpha band (8–13 Hz) divided by the total power generated in that interval. As utilized elsewhere [4], [44], [45], [46], this metric seeks to eliminate variance resulting from non-brain-based factors (e.g., degradation of electrode contact) that occur during all-night EEG recordings. Moreover, this process facilitates an aggregate analysis across two experimental nights in each of thirteen subjects. To control for the degree to which alpha power signifies wakefulness in each individual (i.e., the subject's native alpha generation [47]), this measure was normalized to the corresponding value derived from a baseline period of eyes-closed wakefulness on the same night.

When a broader range of EEG oscillatory activity was considered (Fig. 4), the power at frequencies of 0.5 Hz to 25 Hz (in steps of 0.5 Hz) was estimated using Bartlett's method (2-second segments) and the Goertzel algorithm [48]. The quantities derived from occipital electrodes O1 and O2 were averaged for subsequent analysis. As before, the power spectral density was normalized to a baseline waking spectrum. To facilitate meaningful comparison across frequencies, which have different dynamic ranges, power values were log-transformed [42] and divided by the standard deviation of the log-spectrum observed during quiet NREM sleep (absent sound presentation) on the same night. When the power in the slow/delta band (0.5–4 Hz) and alpha band (8–13 Hz) were considered as absolute, as opposed to relative, measures, the Bartlett spectra were integrated in the corresponding ranges and the resulting power values were standardized in the manner just described.

### Statistical analysis

The influence of EEG spectral content on sleep fragility was interrogated using survival analysis [49]. Each sound series defined a distinct risk period (a “lifetime”) during which sleep could be disrupted—maintenance of sleep constitutes survival, disruption of sleep, a failure.

Only sound series that were preceded by three contiguous 30-second epochs of the same sleep stage and terminated in a sound-induced arousal were used for analysis. (An arousal was judged to be evoked from stimulation if the arousal occurred during the sound or within 5 seconds from its conclusion.) Among these sound series, 109 out of 724 in NREM and 45 out of 267 in REM were right-censored, meaning that sound presentation ended before arousal occurred.

In this paradigm, sleep stability, a function of loudness, describes the probability of tolerating sounds of any given intensity. Sleep fragility, the stability curve's complement, describes the probability of *disruption* due to sounds of any given intensity.

The effect of EEG spectral features on sleep fragility was evaluated with a Cox proportional hazards regression model. The model was stratified across subjects in order to account for individual differences in noise tolerance. Since our loudness scale grew in discrete, 5 dB increments, we employ the exact-partial likelihood method to handle multiple arousals at each sound intensity [50]. A categorical “stage” covariate was also included to control for the conventional measures available to characterize sleep depth.

When the power at distinct frequencies through 25 Hz were tested independently, *p*-values for each frequency were adjusted using the Holm-Bonferroni method for multiple comparisons [51].

## Supporting Information

Figure S1 **Alpha power is stable for minutes.** This plot shows an unbiased estimate of the autocorrelation function of relative spectral content in the alpha band (8–13 Hz) measured in 10-second intervals (depicted smoothed in Figure 1A). The autocorrelogram portrays the correlation of alpha content with its subsequent values for a range of lags. The trajectory used for this figure transcended multiple sleep stages, thus portraying the global stability of alpha content that might be observed at an arbitrary time of night.
(PDF)

Results S1 Supplementary Results.
(PDF)
