# Supplementary figures and images for: Covert Waking Brain Activity Reveals Instantaneous Sleep Depth
Source: PLoS One. 2011 Mar 3;6(3):e17351. doi: 10.1371/journal.pone.0017351 (PMC3048302; doi:10.1371/journal.pone.0017351)

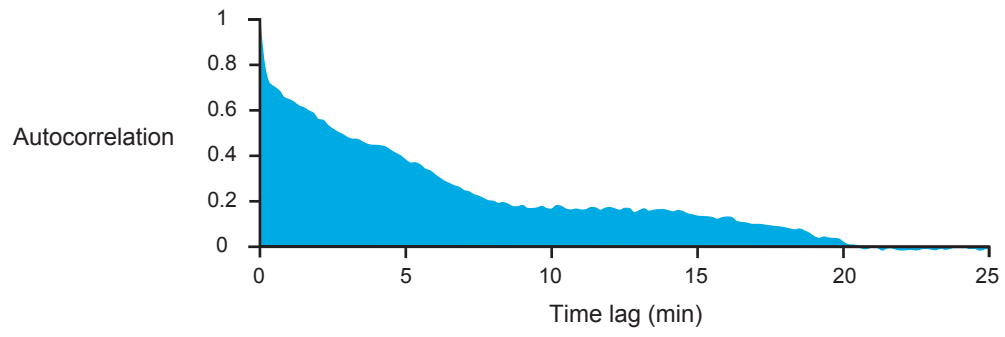

Figure S1

Supplement: Figure S1 — Alpha power is stable for minutes. This plot shows an unbiased estimate of the autocorrelation function of relative spectral content in the alpha band (8–13 Hz) measured in 10-second intervals (depicted smoothed in Figure 1A). The autocorrelogram portrays the correlation of alpha content with its subsequent values for a range of lags. The trajectory used for this figure transcended multiple sleep stages, thus portraying the global stability of alpha content that might be observed at an arbitrary time of night. (PDF) [file pone.0017351.s001.pdf]
