# Supplementary material for: Covert Waking Brain Activity Reveals Instantaneous Sleep Depth
Source: PLoS One. 2011 Mar 3;6(3):e17351. doi: 10.1371/journal.pone.0017351 (PMC3048302; doi:10.1371/journal.pone.0017351)
Supplement: Results S1 — Supplementary Results. (PDF) [file pone.0017351.s002.pdf]

Covert Waking Brain Activity Reveals Instantaneous Sleep Depth

Scott M. McKinney, Thien Thanh Dang-Vu, Orfeu M. Buxton, Jo M. Solet, and Jeffrey M. Ellenbogen*

SUPPLEMENTARY RESULTS

To shed further light on the timescale over which alpha power might be capable of predicting sleep fragility, we studied the natural trajectory of alpha power during an entire night of sleep from one representative subject, as shown in Figure 1A. Here we used data from the quiet night of sleep, free of sound presentation.

The representation of Figure 1B conveys the magnitude of relative alpha power variability over the course of the night. To address the stability of such a measure from one moment to the next, we employed autocorrelation. The autocorrelation function shows the correlation of a time series with its future values at any given latency. Figure S1 shows an unbiased estimate of the autocorrelation function for the relative alpha power trajectory shown in Figure 1A, for lags up to 25 minutes long. This plot portrays the persistence, or inertia, of EEG alpha content from one observation to the next, understanding that stage transitions or awakenings occur many times throughout the night. Although our protocol involving frequent sound stimulation only permitted examination of latencies up to four minutes long, this information from the baseline night suggests that prediction of sleep fragility based on alpha power is not likely valid over time horizons greater than five minutes.

* To whom correspondence should be addressed. E-mail: jeffrey_ellenbogen@hms.harvard.edu
